# Evidence of perpetration of intimate partner violence among HIV-positive couples: a systematic scoping review protocol

**DOI:** 10.1186/s13643-019-1051-3

**Published:** 2019-07-03

**Authors:** Felix Apiribu, Busisiwe Purity Ncama, Elizabeth Joseph-Shehu

**Affiliations:** 1 School of Nursing and Public Health, College of Health Sciences, University of KwaZulu-Natal, Desmond Clearance Building, Howard College Campus, Durban, 4001 South Africa; 2 Department of Nursing, Faculty of Allied Health Sciences, College of Health Sciences, Kwame Nkrumah University of Science and Technology, Kumasi, Ghana; 3 Department of Nursing, Faculty of Health Sciences, National Open University of Nigeria, Jabi, Abuja, Nigeria

**Keywords:** Intimate partner violence, HIV/AIDS, Couples, Disclosure, Behaviour, Low- and middle-income countries

## Abstract

**Background:**

Human immunodeficiency virus (HIV) infection and intimate partner violence (IPV) remain highly sensitive areas that have issues to do with stigmatization in many African countries. Despite the fact that there are several studies on the prevention of HIV, the prevalence of HIV in many African countries is still high. Literature shows that prevention of intimate partner violence is key in the spread of HIV infection. This study will focus on evidence of experiences of HIV positive couples with intimate partner violence and the types of violence experienced. This scoping review will map information about intimate partner violence in low- and middle-income countries as well as other countries with intimate partner violence. There is the need to review these studies on HIV positive couples with intimate partner violence to establish gaps and identify where primary research is necessary. The purpose of this study will be to explore evidence of experiences of HIV-positive couples with IPV and the types of violence experiences by HIV-positive couples.

**Methods:**

This scoping review will involve electronic databases, which will include academic search premier, CINAHL, PsycARTICLES (EBSCO), PsycINFO (EBSCO), ScienceDirect, PubMed, MEDLINE (EBSCO) and Google Scholar. The study will be conducted in two stages: the first stage will map out the studies descriptively while the second stage will map the additional inclusion criteria of quality assessment. Two independent reviewers will undertake the data extraction. Relevant outcomes of the studies will be analyzed thematically using NVivo computer software.

**Results:**

Results on the evidence of the experiences of HIV-positive couples with partner violence will be coded independently by the authors. Thereafter, the authors will critically cross-examine the relationship of the research questions to the emerging themes from the selected articles.

**Conclusion:**

The authors hope to find studies on intimate partner violence among HIV-positive couples to establish gaps where primary research will be necessary.

**Systematic review registration:**

PROSPERO CRD42017062190

## Background

Intimacy issues in relationships are normally very sensitive and secretive especially when they are related to human immunodeficiency virus (HIV) disclosure. Although a lot of work has been done to either minimize it or eliminate it completely as much as possible, fear, ignorance and discrimination regarding HIV continue to exact profound human costs like abusive treatment and violence [4, 16, 18]. Spangenberg et al. identified that 46% of mothers of sick newborns in Ghana reported some form of violence. Women who are HIV positive are at risk of experiencing the same forms of violence as other women in the population but in a severe form because of their status [17].

There is increasing evidence suggesting that HIV-positive women and probably men in many African countries may be at increased risk of experiencing intimate partner violence (IPV) following disclosure of their status to their partners [3, 12, 14]. These problems relating to IPV are, however, more likely to be exacerbated in resource-poor settings like Ghana, where discrimination and stigmatization are high [2, 3]. HIV-positive couples in such areas are at risk of being ostracized, denied some essential services, criticized, thrown out of their homes, divorced and/or blamed for bringing disease into the family or into the community [15]. Shamu et al. further reported that 40.5% of HIV-positive pregnant women in Zimbabwe experienced IPV after disclosure of their HIV-positive status to their partners. HIV-positive women are at increased risk of experiencing IPV following disclosure of the partner’s status to each other [1, 10]. The fear of IPV (physical, sexual, psychological and spiritual abuse) may decrease HIV-prevention behaviours [5, 8, 15] such as disclosure of seropositive status to partners, which may lead to the spread of the infection. It may also prevent HIV-positive couples from staying on lifelong antiretroviral treatment and prevention of mother-to-child transmission [6, 7, 13]. Violence against HIV-positive women also has a consequential influence on status disclosure, which is vital to HIV prevention and partner support in accessing preventive services [8, 9].

Intimate partner violence by men against their partners is one of the most glaring indicators of women’s lack of empowerment [3, 10]. There is often the need for the collection of both victimization and offending data that question both partners. This would allow for an examination of the association between status compatibilities and intimate partner violence and victimization and perpetration, as well as mutually combative behaviour between intimate partners [11].

There is scarcity of evidence on the most effective ways of reducing IPV and abuse among persons living with HIV (PLWHIV) in most sub-Saharan African countries [14] which are low- and middle-income countries. Therefore, the need to review studies on HIV-positive couples with intimate partner violence to establish gaps and identify where primary research is necessary. Hence, this study will explore evidence of experiences of HIV-positive couples with IPV and the types of violence experiences by HIV-positive couples.

### Research objectives

The main aim of the review is to explore evidence of intimate partner violence among human immunodeficiency virus (HIV)-positive couples.

The specific objectives of the study include the following:

1. To review evidence on the types of intimate partner violence among HIV couples
2. To review evidence on the experiences of HIV couples with intimate partner violence

The study will provide answers to the following questions:

1. What are the types of intimate partner violence among HIV couples?
2. What are the experiences of HIV couples with intimate partner violence?

## Methods/designs

This study protocol was registered and published with the International Prospective Register of Systematic Reviews (PROSPERO) with registration number CRD42017062190.

### Search strategy and study selection

This scoping review will involve electronic databases which will include PsycARTICLES (EBSCO), PsycINFO (EBSCO), ScienceDirect, PubMed, MEDLINE (EBSCO) and Google Scholar. The search strategy will include all studies that addressed evidence of experiences of HIV-positive couples with partner violence. All peer-reviewed studies and grey literature that addresses the research questions will be selected in the study. Only articles published in English between 2007 and 2018 will be used. The reason for limiting this study to between 2007 and 2018 is due to lack of resources. The literature search results will be uploaded to EndNote X8. The EndNote X8 software will be used to find and remove duplicates. The search will be conducted over 4 months.

### Criteria for inclusion

- ➢ Articles published in the English language
- ➢ Literature published from January 2007 to December 2018
- ➢ Articles that report on experiences of couples with HIV
- ➢ Evidence from published relevant literatures
- ➢ Studies reporting on experiences of intimate partner violence
- ➢ Review articles including systematic reviews, meta-analysis, scoping reviews, peer-reviewed journal articles and rapid reviews
- ➢ Grey literature sources such as documents from government and non-governmental organizations and academic dissertations
- ➢ All types of study designs such as cohort studies, cross-sectional studies, qualitative studies, quantitative studies, randomized control trial studies, quasi-experimental study designs and pilot studies

### Exclusion criteria

- ➢ Intervention that does not include any form of intimate partner violence
- ➢ Studies not focusing on experiences of HIV-positive couples with intimate partner violence and approaches to reducing its prevalence
- ➢ Studies reporting on other infections other than human immunodeficiency virus/acquired immune deficiency syndrome (HIV/AIDS)
- ➢ Non-English publications
- ➢ Articles published before January 2007 and after December 2018
- ➢ Articles not focusing on HIV-positive adults in intimate relationship.

Keywords search (Table 1) will be combined into a phrase including Boolean (AND, OR) terms, as follows: (intimate partner violence AND HIV/AIDS OR couples OR disclosure OR behaviour OR low and middle income countries).

**Table 1:** A PICOS framework for determination of the eligibility of the review question

Criteria	Determinants
Population	The population of the study will be individuals in intimate relationship and living with HIV
Interventions	Intimate partner violence
Comparisons	None
Outcomes	Promote positive relationship among HIV couples, prevent the spread of HIV and improve quality of life (general well-being and health)
Study settings	Low- and middle-income countries are the focus. Owing to the scarcity of literature on intimate partner violence, we proposed not to limit the scope of this review by study setting. The rationale for this is to have a good number of publications globally for comparison

Updated records of the number of publications identified during each session of the literature search will be kept using the information.

### Data extraction

Numerical summary and thematic analysis will be employed to extract background information from the selected studies. In order to answer the research questions as guided by population, interventions, comparison and outcome (PICOS) (Table 1), the reviewers will collectively design a data-chronicling form to determine the text words, and themes to include and extract. They will also collectively develop the data-charting form.

- ➢ Information and data relevant to answer the research questions will be determined by the reviewers collectively
- ➢ Update recording of data relevant to the study
- ➢ Relevant data will be extracted from all the eligible studies by two independent reviewers in duplicate

Data to be extracted include (i) author (s) names, (ii) year of publication, (iii) study design and/or methodology, (iv) study population, (v) intervention(s), (vi) study setting, (vii) aim of the study, (viii) geographic location of the study and (ix) conclusions.

**Fig. 1:**
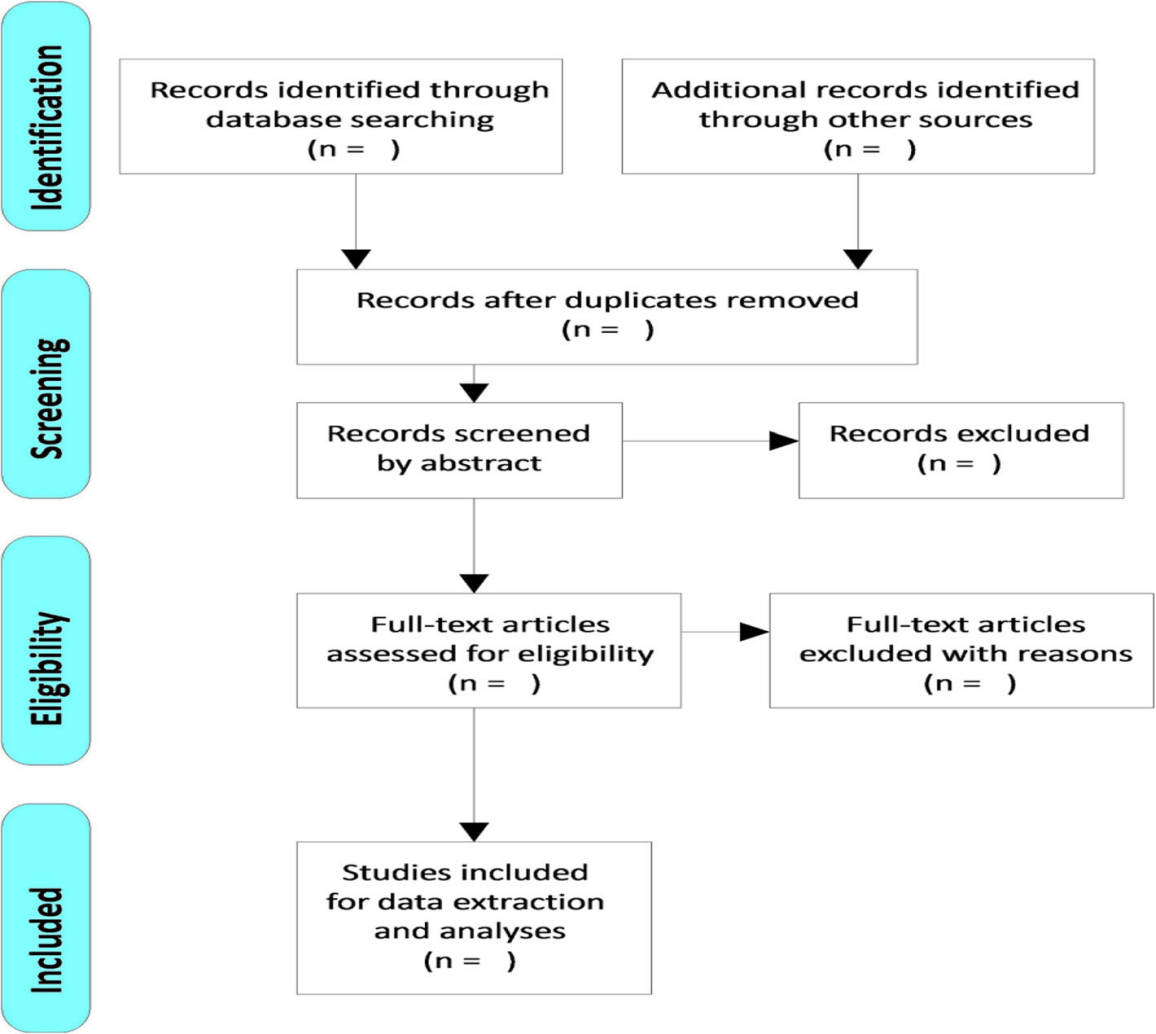
A flow chart showing phases of the literature search for extraction of the most specific literature for the review

Information specific to the evidence of experiences of HIV-positive couples with partner violence will be extracted using phases of the literature search for extraction of the most specific literature for the review as seen in Fig. 1 below.

- ➢ If necessary, a third reviewer will be consulted in order to reach consensus
- ➢ The researchers will collectively carry out a thematic analysis to extract relevant outcomes using NVivo software

### Collating, summarizing and communicating results

The main aim of this review is to scope for existing evidence of the experiences of HIV-positive couples with partner violence and summarize the findings as presented across the articles reviewed. The research team will meet to carry out a thematic analysis of the data collected and provide an overview of all data collected. This process will include the following steps;

- Step 1: Examine the bibliographic details, study designs, number of participants, study setting and funding sources of all included studies
- Step 2: Results on the evidences of the experiences of human immunodeficiency virus (HIV)-positive couples with partner violence will be coded independently by the authors. All the authors will then come together and interrogate the resultant themes, critically look at their relationship to the set research questions
- Step 3: The research team will examine the implications for future research, practice and policy based on the main aim of the study

### Patient and public involvement

Patients and/or the public were not involved in this study.

## Discussion/conclusion

This study will generate evidence that will help to describe experiences of human immunodeficiency virus (HIV)-positive individuals/couples with intimate partner violence. Recent improvements in antiretroviral therapy, coupled with nutritional supplementation, have contributed to longevity of life of persons living with HIV (PLWHIV). Mortality and morbidity are now low in HIV populations in low- and middle-income countries while campaigns have further slowed the spread of the virus. However, these efforts are being hindered by non-disclosure of HIV-seropositive status by some couples for fear of being victims of intimate partner violence. Good intimate partner relationship can improve the quality of life of HIV-positive couples and increase their life expectancy. IPV prevention can contribute immensely on the prevention of HIV/AIDs.

We are not aware if there are any empirical studies or reviews that have been done in sub-Saharan African countries that answer the research questions outlined in this protocol. Therefore, this study will include all studies from anywhere in the world that address the population of interest, intervention and any/or all the outcomes listed in Table 1.

Findings from this study will contribute to the body of knowledge on intimate partner violence among HIV-positive individual/couples which will have a positive impact on research, practice and policy. Reduction or elimination of intimate partner violence should help improve the quality of life of HIV-positive couples and increase their life expectancy. The findings from the study will provide an insight on the possible means of reducing IPV and abuse among PLWHIV.

The proposed systematic scoping review results will generate findings that will describe the experiences of HIV-positive couples with partner violence, and the types of violence these populations experienced. The findings from this study will help in identifying gaps in literature on the care of the HIV-positive couples with partner violence. It will also enhance HIV prevention that can be used in education, clinical practice and in making public health policies on HIV and AIDS prevention. The gaps identified will help outline areas for preventive strategies to stimulate cordial coexistence among couples in the low- and middle-income countries. However, there is no data on intimate partner violence among HIV-positive couples in low- and middle-income countries. A number of studies have been on HIV and stigmatization among both men and women.

## Data Availability

Since this manuscript is a protocol for a scoping review, there are no data to share. However, when the study is complete we would be happy to share the dataset.

## Abbreviations

AIDS: Acquired immune deficiency syndrome
HIV: Human immunodeficiency virus
HIV/AIDS: Human immunodeficiency virus/acquired immune deficiency syndrome
IPV: Intimate partner violence
PICO: Population, interventions, comparison, and outcome
PLWHIV: Persons living with the human immunodeficiency virus
